# Medical News and Items

**Published:** 1877-03

**Authors:** 


### Medical News and Items.

Rush Medical College.—The thirty-fourth Annual Commencement Exercises of Rush Medical College were held in the new college building, corner of Wood and Harrison streets, Wednesday evening, February 21, 1877. The large amphitheatre was crowded by nearly a thousand physicians and friends of the college. Many hundreds were unable to gain admittance to the overcrowded audience room. The presence of ladies and excellent music contributed largely to the enjoyment of the occasion. After prayer, by the Rev. Francis L. Patton, D.D., of the Presbyterian Theological Seminary, President J. W. Freer, M.D., conferred the degree of Doctor in Medicine upon one hundred and three gentlemen who have complied with the requirements necessary to obtaining the degree. From the appended list of graduates furnished for publication, *fifteen* significant erasures indicate that as many ambitious young hearts were saddened by not bearing their examinations to the satisfaction of the Faculty's demands.

*Graduates' Names.*—Eugene Savillian Atwood, Silas Addison Austin, Charles Rucker Aiken, Abraham Ashbaugh, Macaulay Arthur, John Wesley Andrews, George Edward Brown, Vernon Roe Bridges, William Thomas Belfield, William Harden Boals, George Henry Barney, William Allds Burnham, Herbert Roderick Bird, John Charles Bryan, Thomas Davis Baird, Benjamin Hurst Bean, John Wesley Clendening, James St. Clair C. Cussins, Robert Cottington, Charles Augustine Cromett, Charles Edward Clingan, Andrew M. Crawford, Charles Edwin Caldwell, Irving Le Roy Cutler, Charles Peter Caldwell, William Joseph Conan, George Patrick Cunningham, Daniel Chambers Darroch, Levi Dixon, William Morris Evans, James Marcus Everett, Frank William Epley, William Robert Freek, Dexter Boylston Farnsworth, John Welton Fisher, George Washington Gurnea, George Frederick Gay, William Martin Graham, William Orlando Harland, Edwin William Hunter, Charles Addison Hayes, Hamilton Worth Hewit, Sylvester Clay Ham, Newell Hiram Hamilton, Joseph Mosher Heller, Virgil Eusebius Hestwood, Lyman Drake Jackson, William Henry Jennings, Jacob C. Joralemon, Charles Ludwig Koch, Henry Charles Kerber, Frederick Simon Luhman, Edwin J. Lewis, Leslie Coulter Lane, John Wesley La Grange, James Lawless, John Hinton Lowry, Elmer Fremont Latta, Charles Adolph Luscher, Ottul Klaranus Lindboe, William Herbert Lynn, James McDougle, Joseph Constantine McMahan, John Randolph McCluggage, Theodore Warner Morse, John Wellington Morton, Freeman C. Mason, Thomas

Coleman Malone, Hosea Fountain C. Miller, Jesse Marion Mathes, William Netter, Edwin McLean Northcott, Frederick Robert Nitzsche, James Henry Plecker, George H. Peters, William F. Quirk, Frank Darlington Rathbun, Hugh Alexander Rose, Joseph Bently Rogers, John Allen Russell, Albert Bird Royal, James Lee Reat, Milo Wakely Scott, Horace Woodbridge Smith, Farquhar Stuart, Oliver Thomas Shenick, Thomas Patrick Shanahan, Myron Arthur Tibbits, James Lewis Taylor, Merritt Walter Thompson, William Hull Ten Brook, William Treacy, Ryan Teeguarden Van Pelt, Clark Wesley Voorus, Charles Myron Willis, Clarence Scott Wells, Winfred Wylie, William Henry Washburne, Joel Wallace Whitmire, Robert Henry Williamson, Charles Zuppann. *Ad Eundem*—James Degman Reynolds, M.D., Julius Otto, M.D., Charles Peter Caldwell, M.D.

The President of the Faculty *mare mojorum* followed the bestowal of diplomas with his brief charge to the class. He warmly commended their high attainments, unswerving fidelity to the conscientious use of their time during the past five months, and uniform courtesy to their teachers, congratulating them upon having successfully passed an unusually severe set of examinations.

At the conclusion of this address, Dr. Chas. A. Hayes, on behalf of the class, delivered the class valedictory, thanking the Faculty for their teachings, and promising many good things for the future.

After Dr. Hayes, Prof. Jos. P. Ross proceeded to pronounce the valedictory address of the occasion. We regret our inability to reproduce this address in full, for it was one replete with wise advice to the aspiring young doctors before him, and was a farewell word, eminently appropriate to the termination of a protracted and finishing course of instruction in medicine.

After the benediction and music, the large audience wandered through the capacious building, subjecting every part of it to inspection and criticism.

A more enjoyable occasion in medical matters in Chicago, it has scarcely ever been our good fortune to participate in. The happy faces of the graduates, the settled, comfortable satisfaction expressed in the countenances of the Faculty and Trustees over their elegant new home, and the general *bonhomie* of the audience, combined to impress every one present that it was good for them to be there.

---

At the *Woman's Hospital Medical College* the following students have been graduated: Blanche O. Burroughs, Aurora, Ill., Louisa Dawson, Evansville, Wis., Ellen Von Rolshausen, Chicago, Jennie E. Tarbox, Freeport, Ill. They received their diplomas at the commencement exercises in the First Methodist Church, February 27.

---

The Library of the Chicago Medical Press Association will, on March 1st, be removed from the Academy of Sciences to a spacious room, the use of which is generously donated by Dr. E. Ingals, at 188 South Clark street. This location, in the very centre of the city, is easily accessible from each division, so that even the busiest practitioner may avail himself of the privileges offered by the Association.

The Library, as soon as the hurry and confusion of moving are over, will be kept open by a regular attendant at least six hours each day for the use of members. In our next issue will appear a card from the Librarian regarding the condition and needs of the Library. We will say now, however, that any donations of books addressed to the Library at the above number will be duly and gratefully received.

---

[The following bill, introduced into our State Legislature by Mr. Joslyn, January 8, 1877, has been ordered to a third reading. Pending this order, a new, and much more comprehensive bill has been introduced, the daily papers inform us. While this bill is ostensibly calculated to protect the *dear* public (which never loses an opportunity to belittle educated physicians and to run mad over blatant quacks,) it will result in driving charlatans in swarms to the under-fed, half-clothed colleges, whose chief occupation is to sell diplomas. Armed with such sheepskins, these vermin can sit under your noses and ours and defy educated physicians to purify the ranks of the profession again. Bills of this sort do no good. Medical matters can be legistated into optimism *only* by controlling the colleges, or by exacting from them the proper qualifications in graduates before bestowing diplomas. First class colleges need no such legislation. "Diploma mills," as the great Cowling calls them, would be ground to powder by it, and *they* are what legislatures should severely turn their attention to.—Ed.]

A Bill for an act to Regulate the Practice of Medicine and Surgery in the State of Illinois.

Section 1. *Be it enacted by the People of the State of Illinois, represented in the General Assembly*, That it shall be unlawful for any person within the limits of said State of Illinois who has not attended at least two full courses of instruction and graduated in some chartered school of medicine, either in the United States or some foreign country, and is not a person of good moral character, to practice medicine in any of its departments, or perform any surgical operations for reward or compensation, or attempt to practice medicine or prescribe medicine or medicines, or perform any surgical operations for reward or compensation within the State of Illinois.

§ 2. Any person living in the State of Illinois, or any person coming into said State, who shall practice medicine, or attempt to practice medicine, in any of its departments, or perform or attempt to perform any surgical operation upon any person within the limits of said State, in violation of section one of this act shall, upon conviction thereof, be fined not less than fifty dollars ($50) nor more than one hundred dollars ($100) for such offense, and upon conviction for a second violation of this act, shall in addition to the above fine, be imprisoned in the county jail of the county in which such offense shall have been committed, for the term of thirty days; and in no case wherein this act shall have been violated, shall any person so violating receive any compensation for services rendered.

§ 3. Any person who fails or neglects, on or before the first day of October, 1877, to file in the office of the clerk of the circuit court of the county in which he resides or keeps his office, a certificate or diploma of some chartered college of medicine, that he has attended at least two full courses, and graduated at such college, shall not be permitted in any court of this State to sue for or recover any compensation for his services, advice or attendance as a physician or surgeon; and the failure to file a certificate or diploma as above provided, shall be *prima facie* evidence that he has not attended or graduated at any school of medicine.

§ 4. Any person filing a certificate or diploma as provided in section three (3) of this act, shall attach an affidavit thereto that the same is true and genuine: *Provided*, that any person now practicing medicine or surgery may be examined by the faculty of either of the following named medical colleges: Rush Medical College of Chicago, Chicago Medical College, Chicago, Hahnemann Medical College of Chicago, Bennett Medical College of Chicago, Missouri Medical College of St. Louis, St. Louis Medical College of St. Louis, Homœopathic Medical College of Missouri at St. Louis, American Medical (Eclectic) College of St. Louis, or the Louisville Medical College at Louisville, Kentucky; and, if found by such faculty competent to practice medicine or surgery, said faculty shall grant such persons diplomas without the courses of instruction provided for in section one (1) of this act."

---

A correspondent to the *N. Y. Med. Record* draws this picture of Prof. Billroth: "A profound pathologist, an accurate anatomist, an operator bold to the verge of rashness, an easy conversational lecturer, an accomplished linguist, a good blackboard draughtsman, are qualities not every day to be found combined in one who, during the most severe and tedious operations, preserves an amiability and unpretentiousness which makes his presence a companionship to the youngest assistant. Nor does one often find the strength and endurance of a blacksmith uniting these qualities on the one hand to a distinguished social reputation as a composer and pianist on the other. A

combination of qualities like this, in one so favorably circumstanced, could hardly fail in achieving the popularity and success which Prof. Billroth has accomplished."

---

Table Showing the Ages at which Marriages take place in each Sex. Taken from the Reports of the Board of Health of Philadelphia, for fifteen years, 1861–1875.

| | Und'r20 | 20 to 25 | 25 to 30 | 30 to 40 | 40 to 50 | 50 to 60 | 60 to 70 | 70 to 80 | 80 to 90 | Total. |
|---|---|---|---|---|---|---|---|---|---|---|
| Whole No. Males........ | 417 | 32951 | 28792 | 17515 | 5294 | 1783 | 498 | 94 | 9 | 87353 |
| Whole No. Females.... | 17604 | 40467 | 16624 | 9504 | 2546 | 524 | 80 | 3 | 1 | 87353 |
| Per cent. Males ........ | 00.48 | 37.72 | 32.96 | 20.05 | 6.06 | 2.04 | 0.57 | 0.11 | 0.01 | 100.00 |
| Per cent. Females...... | 20.15 | 46.33 | 19.03 | 10.88 | 2.914 | 0.60 | 0.092 | 0.003 | 0.001 | 100.00 |

It will be noticed by examining the above table of eighty-seven thousand three hundred and fifty three marriages, that "the chances of females being married before the age of twenty years, are as *one to five* of all the probabilities that they will ever marry. At the age of twenty years about *one-fifth* of all their chances are gone. At twenty-five, about *two-thirds*, and at thirty, nearly *six-sevenths* of all their probabilities are lost."

Man's chances are scarcely lessened at the age of twenty; at twenty-five, about *three-fifths* of their chances remain ; at thirty, about *seven-tenths* of their chances are gone.

The number of births in Philadelphia, for the same period, amounted to two hundred and fifty-eight thousand six hundred and twenty-one, of which one hundred and thirty-five thousand eight hundred and four were males, one hundred and twenty-two thousand eight hundred and seventeen females. There were eleven thousand eight hundred and thirty-six still births, or one to every 21.8 births. One woman out of every 104.1 in labor gave birth to more than one child. Thirty-two women during the period mentioned, gave birth to triplets.

---

Harvey Demonstrating the Circulation of the Blood.—The great scarcity of the original engraving of Harvey demonstrating the circulation of the blood to Charles the First of England, and published in 1851 by Lloyd Brothers, of London, has induced Mr. H. Wood, Jr., of 826 Broadway, N. Y., to issue a photograph of the same. It is 7 by 9 inches in size, and is admirably well executed. By comparing it with the original engraving, it can be readily seen that in sharpness of detail it is everything which could be desired. As the London engraving is now out of print, and therefore very difficult to obtain, this excellent photographic copy is likely to become a very popular picture for the office.

---

Bust of Desault.—On the 15th of October last there was a grand celebration in the city of Lure, in France, on the occasion of the unveiling of the bust of the illustrious surgeon Desault, the bust being the work of the artist Iselin.

Desault's name requires no comment for the medical profession of America. He was the grand teacher of such great men as Bichât, Dupuytren, Larrey and Chopart, the man who laid broad and deep the foundation of the science of surgery as practiced in the nineteenth century; the son of a peasant, and yet one whose genius placed him in that proud position which is well described in the eulogy pronounced by the immortal Bichât, his pupil.

---

Ascarides *vs.* Asteroids.—A married lady consulted her physician in regard to a difficulty of the pelvic viscera, having been, during a protracted maidenhood, engaged in the exacting occupation of a teacher in the common schools of this State, which demands, as one of the requirements for a license, a knowledge of anatomy and physiology. She was at no loss to describe the anatomical parts severally affected in good language; however, in an unguarded moment she wandered beyond her domain, and, while referring to a former disease of the rectum, in which *ascarides* played an important part, she declared that since she had got rid of those little *asteroids* she had not had the train of symptoms she had just enumerated.

A new journal is announced from Detroit, the *Detroit Medical Journal*, a monthly. It is made by the consolidation of the *Detroit Review of Medicine and Pharmacy* and *The Peninsular Journal of Medicine.* We are glad to see our friends unite forces upon one Journal; we know it will be a strong one. We are also glad the new periodical is to be issued under the auspices of the Detroit Medical and Library Association. We think we see here the promise of a great medical library for Detroit.

---

*The American Medical Weekly*, of Louisville, has begun the new year as a *bi-weekly.* For the future, Editor Gaillard promises not to quarrel with his neighbors over the way. On the other hand, the *Louisville Medical News* says this promise is due to the fact of its continued drubbing of the *Weekly* and the double-headed medical college, of which it says the *Weekly* is the organ. If our friends, the medical editors of Louisville, can be believed, somebody has been very naughty down there.

---

The fifty-first Annual Report of the Massachusetts Charitable Eye and Ear Infirmary shows that during the year ending Sept. 30, 1876, 8,022 patients were treated in the dispensary, and 431 as house patients, making a total number of 8,453 patients. The out patients are classified as eye patients (5,754) and ear patients (2,268), but no such division is made with the house patients. Still the statistics of the out patients show again the decided preponderance of the external diseases of the eye, the diseases of the lids, conjunctiva, cornea, iris and lachrymal apparatus, numbering 4,170, or 72 per cent. of the total of 5,754. The number of operations performed on the eye is 309, exclusive of the removal of foreign bodies from the cornea and conjunctiva.

---

There are are twelve homœopathic medical journals published in the United States.

